# Impact of poor glycemic control upon clinical outcomes after radical prostatectomy in localized prostate cancer

**DOI:** 10.1038/s41598-021-91310-3

**Published:** 2021-06-07

**Authors:** Hakmin Lee, Seok-Soo Byun, Sang Eun Lee, Sung Kyu Hong

**Affiliations:** 1 Department of Urology, Seoul National University Bundang Hospital, 82 Gumi-ro, 173 Beon-gil, Bundang-gu, Seongnam-si, 463-707 Gyeonggi-do Korea; 2 Department of Urology, Seoul National University College of Medicine, Seoul, Korea

**Keywords:** Urology, Prostate

## Abstract

To evaluate the clinical impact of preoperative glycemic status upon oncological and functional outcomes after radical prostatectomy in patients with localized prostate cancer, we analyzed the data of 2664 subjects who underwent radical prostatectomy with preoperative measurement of hemoglobin A1c within 6 months before surgery. The possible association between high hemoglobin A1c (≥ 6.5 ng/dL) and oncological/functional outcomes was evaluated. Among all subjects, 449 (16.9%) were categorized as the high hemoglobin A1c group and 2215 (83.1%) as the low hemoglobin A1c group. High hemoglobin A1c was associated with worse pathological outcomes including extra-capsular extension (HR 1.277, 95% CI 1.000–1.630, *p* = 0.050) and positive surgical margin (HR 1.302, 95% CI 1.012–1.674, *p* = 0.040) in multi-variate regression tests. Kaplan–Meier analysis showed statistically shorter biochemical recurrence-free survival in the high hemoglobin A1c group (*p* < 0.001), and subsequent multivariate Cox proportional analyses revealed that high hemoglobin A1c is an independent predictor for shorter BCR-free survival (HR 1.135, 95% CI 1.016–1.267, *p* = 0.024). Moreover, the high hemoglobin A1c group showed a significantly longer incontinence-free survival than the low hemoglobin A1c group (*p* = 0.001), and high preoperative hemoglobin A1c was also an independent predictor for longer incontinence-free survival in multivariate Cox analyses (HR 0.929, 95% CI 0.879–0.981, *p* = 0.008). The high preoperative hemoglobin A1c level was independently associated with worse oncological outcomes and also with inferior recovery of urinary continence after radical prostatectomy.

## Introduction

Diabetes mellitus (DM) is one of the most prevalent and disabling chronic diseases in many developed countries, including the United States^1^. DM is a well-known risk factor for several serious diseases such as stroke, heart attack, vision impairment, peripheral vascular disease, and even depression^2^. Moreover, DM is also associated with cancer development and aggressive clinical behaviors^3^. However, the relationship between DM and prostate cancer (PCa) seems to be more complicated^4^. Unlike most other cancers, which usually have positive relationship between DM and the development of cancer, several previous meta-analyses have shown an inverse relationship between DM and PCa^5–6^. Previously, we investigated the impact of DM on oncological outcomes in patients treated with radical prostatectomy (RP) for localized PCa^7^. However, we could not find any significant associations between DM and clinico-pathologic outcomes after RP. Conversely, we observed that poor glycemic control, which was represented by hemoglobin A1c (HbA1c) level, was significantly associated with some adverse pathologic features, including high Gleason score and extra-capsular extension among the patients with DM. Subsequently, another study reported similar findings after analyzing the Shared Equal Access Regional Cancer Hospital database after surgery^8^. In their study, HbA1c tertile was predictive of a higher pathologic Gleason score (*p* = 0.001). However, their study was limited due to the small sample size (n = 247). Despite the interesting results of these two studies, they only analyzed the impact of preoperative glycemic control in patients who were already diagnosed with DM but not in those who did not have prior DM diagnosis. As several other studies also showed that the preoperative glycemic control was associated with worse prognosis in other malignancies, including colorectal and liver cancers, the impact of glycemic control can also be prognostic in patients with PCa regardless of previous DM history^9–10^. Therefore, we tried to evaluate the clinical impact of preoperative glycemic control on the oncological and functional outcomes in patients who were treated with RP for localized PCa.

## Materials and methods

After obtaining approval from Seoul National University Bundang Hospital Institutional Review Board, we retrospectively analyzed the data of 2716 subjects who were diagnosed with localized PCa and subsequently treated with RP between January 2010 and December 2018. All analyses were performed in accordance with the guidelines and regulations of our institutional review board and informed consent was waived due to the nature of retrospective analyses and minimal risk to the participants. After the additional exclusion of 52 patients (preoperative androgen deprivation therapy [n = 18], previous pelvic radiation [n = 3], and incomplete information [n = 31]), we finally analyzed the data of 2664 patients. Clinical and pathologic information was retrieved from our prospectively maintained institutional database. HbA1c measurements were routinely performed as part of the preoperative work-up. The history of DM was verified by self-administered questionnaires at the time of admission for surgery, which asked if they had been diagnosed with DM during their lifetime. Patients who were diagnosed with DM before surgery were considered to have a history of DM. The type of surgery (open/robotic/laparoscopic), degree of neurovascular bundle preservation and lymph node dissection were decided by each surgeon’s clinical opinion and/or counseling with patients. The measurement of HbA1c was routinely performed as a part of preoperative work-up for general anesthesia in this study. To determine the optimal cutoff value for HbA1c, the receiver operating curve of HbA1c on BCR was analyzed. Since the HbA1c value of 6.5 ng/dL showed the largest Youden’s index, the cutoff value was set at 6.5 ng/dL. Therefore, patients with HbA1c < 6.5 ng/dL were categorized as the low HbA1c group, while the others were categorized as the high HbA1c group. Pathologic outcomes such as seminal vesicle invasion, an extraprostatic extension of tumor, positive surgical margin, and lymph node invasion were inspected as previously described^7^. BCR was defined as a prostate-specific antigen (PSA) level ≥ 0.2 ng/mL in two consecutive tests as per the guidelines of the American Urological Association ^11^. The follow-up period was defined as months from the date of surgery to the date of the last visit or mortality. After surgery, patients were usually followed up at an interval of 3- to 6-months during the initial two years and yearly thereafter, when there was no evidence of BCR. The functional outcomes after surgery were evaluated using a patient-reported questionnaire, including the Expanded Prostate Cancer Index Composite for Clinical Practice and International Index of Erectile Function (IIEF)-5. The recovery of urinary continence was defined when the amount of pad usage was counted under one pad/day, and erectile dysfunction was defined when the IIEF-5 score was under 22.

To compare the clinical characteristics between the subgroups, chi-square, and Student’s t-tests were performed. Multivariate binomial regression tests were performed to evaluate possible associations of high HbA1c levels with adverse pathological outcomes. To analyze survival outcomes, Kaplan–Meier analysis and Cox proportional hazard model were utilized. All statistical analyses were performed using SPSS software (SPSS 22.0, Chicago, IL, USA). All *p* values are presented as two-sided values, and *p* < 0.05 was considered statistically significant.

## Results

The clinical and pathological characteristics of all patients are summarized in Table 1. The median age was 67.0 (interquartile range [IQR] 62.0–72.0) years and median PSA was 7.4 (5.0–12.4) ng/dL. Among entire patients, 1184 (44.4%) patients had radical prostatectomy with neurovascular bundle preservation. There were 124 (4.6%) patients who had positive lymph node invasion from surgical pathology and 164 (6.2%) patients had salvage therapy after sugery. There were 2215 (83.1%) patients in the low HbA1c group and 449 (16.9%) in the high HbA1c group. When we compared the distribution of clinicopathological characteristics between the two subgroups, the prevalence of DM and hypertension was significantly higher in the high HbA1c group than in the low HbA1c group (all *p* < 0.001). The high HbA1c group also showed significantly unfavorable clinical features in terms of preoperative PSA, biopsy grade group, clinical stage, pathologic grade group, and pathologic stage (all *p* < 0.05). Similarly, adverse pathologic outcomes such as positive surgical margin (*p* = 0.003), seminal vesicle invasion (*p* = 0.032), and extra-capsular extension (*p* = 0.001) were also more prevalent in the high HbA1c group. Multivariate regression analyses showed that HbA1c was independently associated with extracapsular extension (HR 1.277, 95% CI 1.000–1.630, *p* = 0.050) and positive surgical margin (HR 1.302, 95% CI 1.02–1.674, *p* = 0.040), respectively (Table 2).

**Table 1:** Summarization of clinico-pathologic characteristics according to the hemoglobin A1c level.

Median (IQR) or number (percent)	Entire patients (n = 2664)	High HbA1c group (n = 449)	Low HbA1c group (n = 2215)	*p* value
Age (y)	67.0 (62.0–72.0)	68.0 (63.0–72.0)	67.0 (61.0–71.0)	0.171
BMI (kg/${\mathrm{m}}^{2}$)	24.6 (22.9–26.3)	25.1 (23.3–26.7)	24.5 (22.9–26.2)	0.897
Diabetes mellitus	22.3%	75.5%	11.5%	< 0.001
Hypertension	49.0%	59.2%	46.9%	< 0.001
PSA	7.4 (5.0–12.4)	7.9 (5.3–14.0)	7.3 (5.0–12.0)	< 0.001
Prostate volume	33.5 (26.0–42.5)	34.4 (27.0–44.0)	33.0 (26.0–42.0)	0.007
**Biopsy grade group**				0.035
Group 1	28.9%	24.3%	29.8%	
Group 2	31.4%	29.9%	31.7%	
Group 3	18.8%	21.9%	18.1%	
Group 4	16.2%	17.6%	15.9%	
Group 5	4.7%	6.3%	4.4%	
**Clinical stages**				0.202
cT1	53.0%	51.0%	53.4%	
cT2	27.1%	30.5%	26.5%	
cT3	19.9%	18.5%	20.2%	
**Pathologic grade group**				0.028
Group 1	4.6%	5.1%	4.5%	
Group 2	42.4%	36.6%	43.6%	
Group 3	35.7%	36.6%	35.5%	
Group 4	6.5%	7.8%	6.2%	
Group 5	10.8%	13.8%	10.2%	
**Pathologic stages**				0.041
pT2	9.5%	7.4%	9.9%	
≥ pT3	90.5%	92.7%	90.1%	
ECE	31.6%	38.4%	30.2%	0.001
SVI	12.1%	15.2%	11.5%	0.032
PSM	21.2%	26.6%	20.1%	0.003

*IQR*, interquartile range, *BMI* body mass index, *PSA* prostate specific antigen, *ECE* extracapsular extension, *SVI* seminal vesicle invasion, *PSM* positive surgical margin.

**Table 2:** Multivariate analyses for the impact of high HbA1c level on aggressive pathologic outcomes.

	HR	95% CI of HR	*p* value
Pathologic Gleason score (≥ 4 + 4)	1.279	0.976–1.675	0.075
Pathologic Stage (≥ T3)	1.251	0.847–1.849	0.260
Extracapsular extension	1.277	1.000–1.630	0.050
Seminal vesicle invasion	1.114	0.789–1.573	0.541
Positive surgical margin	1.302	1.012–1.674	0.040

*HbA1c* hemoglobin A1c, *HR* hazard ratio, *CI* confidence interval.

Multivariate analyses were adjusted for age, prostatic specific antigen, body mass index, prostate volume, pathologic Gleason score and pathologic stage.

**Figure 1:**
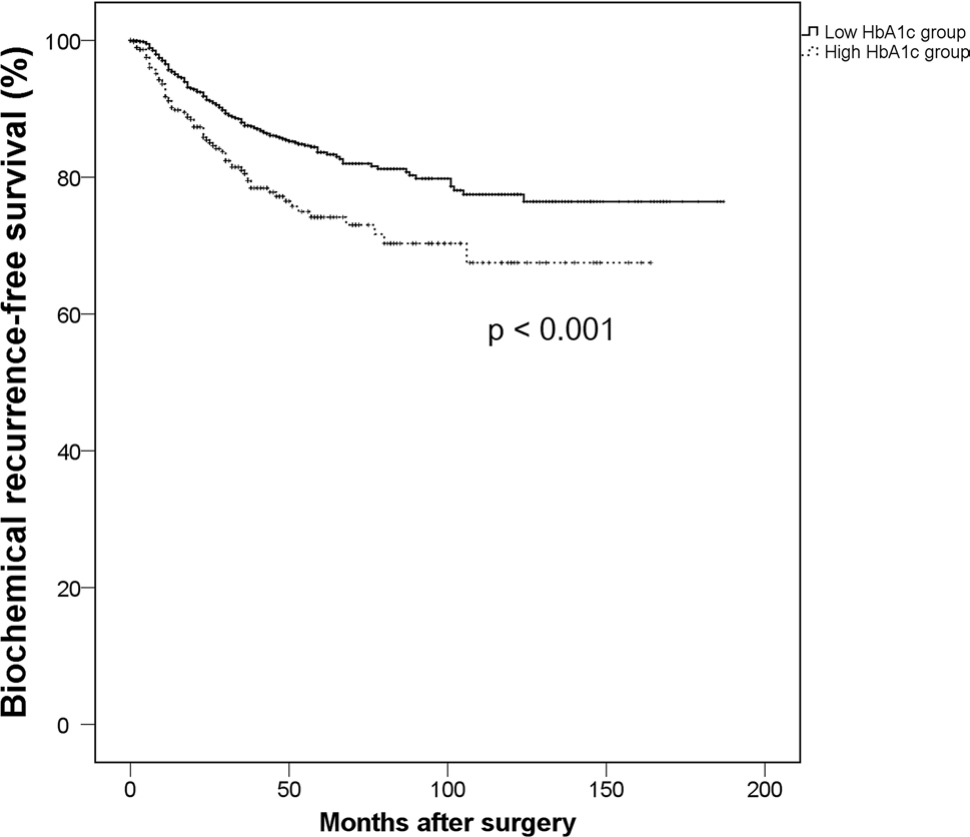
Kaplan–Meier analyses on biochemical recurrence-free survival according to the preoperative HbA1c.

The total medial follow-up time of all subjects was 23 months (IQR, 6.0–47.0). After a median follow-up time of 8 (IQR 6.0–23.0) months, 443 (16.6%) patients developed BCR [329 (14.9%) subjects in low HbA1c group, 114 (25.4%) in high HbA1c group]. Among them, there were 172 (6.5%) patients who had persistent-detectable PSA. The Kaplan–Meier analyses showed that the high HbA1c group had a significantly shorter BCR-free survival than the low HbA1c group (*p* < 0.001) (Fig. 1). The subsequent multivariate Cox proportional hazard analyses revealed that a high HbA1c level was an independent predictor for shorter BCR-free survival (HR 1.135, 95% CI 1.016–1.267, *p* = 0.024), while the preoperative history for DM did not show any statistically significant results (Table 3, Supplementary Table 1).

**Table 3:** Multivariate analyses using Cox proportional hazard model on biochemical recurrence.

	When adjusted by history of DM			When adjusted by HbA1c level		
	HR	95% CI	p value	HR	95% CI	p value
Age	0.978	0.962–0.995	0.013	0.978	0.962–0.995	0.012
BMI	1.006	0.972–1.043	0.719	1.004	0.966–1.043	0.845
History of DM	1.043	0.794–1.371	0.760		Not included	
HbA1c		Not included		1.135	1.016–1.267	0.024
PSA	1.003	1.001–1.005	0.017	1.003	1.000–1.005	0.024
Prostate volume	0.998	0.988–1.008	0.743	0.998	0.988—1.008	0.715
**Pathologic grade group**						
Group 1		Reference			Reference	
Group 2	3.715	0.895–15.416	0.071	3.754	0.905–15.576	0.068
Group 3	18.148	4.429–74.357	< 0.001	18.012	4.397–73.788	< 0.001
Group 4—5	39.749	9.606–164.487	< 0.001	40.121	9.700–165.954	< 0.001
Pathologic stage (≥ pT3)	2.678	1.256–5.707	0.011	2.674	1.255–5.698	0.011
PSM	2.811	2.185–3.618	< 0.001	2.797	2.172–3.600	< 0.001
Lymph node invasion	1.674	0.855–3.277	0.133	1.747	0.894–3.412	0.103

*BMI* Body mass index, *DM* diabetes mellitus, *HbA1c* hemoglobin A1c, *PSA* prostate specific antigen, *PSM* positive surgical margin.

**Figure 2:**
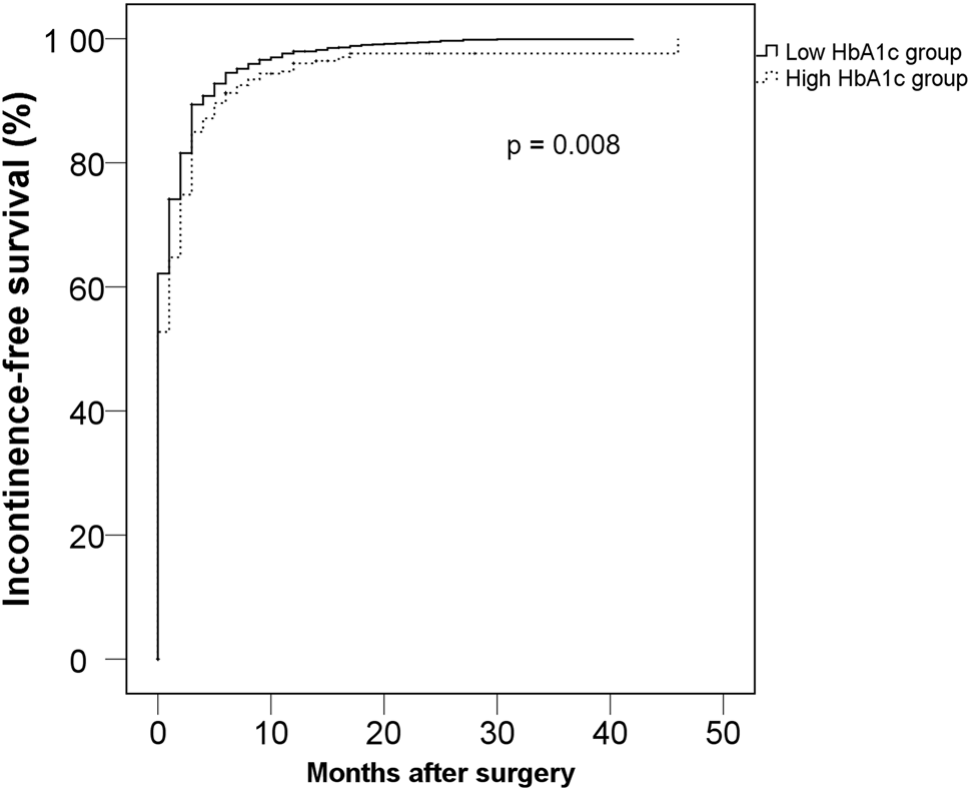
Kaplan–Meier analyses on the recovery of urinary incontinence according to the preoperative HbA1c.

**Figure 3:**
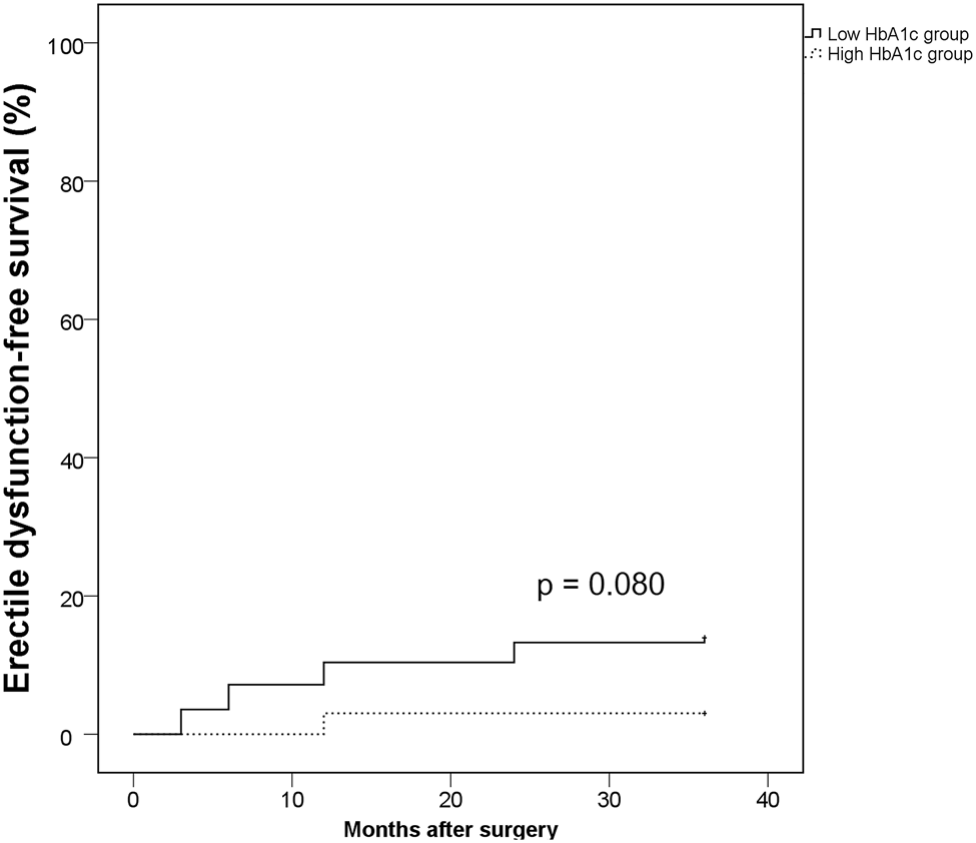
Kaplan–Meier analyses on the recovery of erectile dysfunction according to the preoperative HbA1c.

Among all subjects, 2041 (76.6%) patients completed questionnaires about functional outcomes. Among them, 99 (4.9%) patients still had incontinence after a median follow-up of 31 (IQR, 12.0–51.0) months. The Kaplan–Meier analyses showed that the high HbA1c group showed significantly longer incontinence-free survival (*p* = 0.008) (Fig. 2). Subsequent Cox proportional hazard analysis revealed that a high HbA1c level was an independent predictor for longer incontinence-free survival (HR 0.929, 95% CI 0.879–0.981, *p* = 0.008) when analyzed as a continuous variable. A similar result was observed when HbA1c was adjusted as a categorical variable (HR 0.862, 95% CI 0.765–0.673, *p* = 0.016) (Table 4). Among all patients, 312 (11.7%) patients had no erectile dysfunction before surgery. When we compared the erectile dysfunction-free survival between the high and low HbA1c groups in those patients who were potent before RP, the high HbA1c group showed longer erectile dysfunction-free survival, but this result was not statistically significant (*p* = 0.080) (Fig. 3).

**Table 4:** Multivariate analyses using Cox proportional hazard model on continence-free survival.

	HbA1c as continuous variable			HbA1c as categorical variable		
	HR	95% CI	p value	HR	95% CI	p value
Age	0.992	0.985–0.998	0.016	0.992	0.985–0.998	0.016
BMI	1.002	0.984–1.020	0.853	1.002	0.984–1.020	0.827
PSA	1.000	0.998–1.002	0.972	1.000	0.998–1.002	0.946
Prostate volume	1.000	0.997–1.003	0.935	1.000	0.997–1.004	0.903
HbA1c	0.929	0.879–0.981	0.008	0.862	0.765–0.973	0.016
High pathologic grade group (≥ group 4)	1.098	0.963–1.252	0.162	1.103	0.968–1.258	0.142
High pathologic stage (≥ 3)	1.023	0.879–1.192	0.766	1.024	0.879–1.192	0.765
**Neurovascular bundle preservation**						
None		Reference			Reference	
Unilateral / partial	1.121	0.929–1.354	0.234	1.098	0.904–1.335	0.346
Bilateral	1.184	1.058–1.326	0.003	1.147	1.020–1.290	0.022

*BMI* Body mass index, *DM* diabetes mellitus, *HbA1c* hemoglobin A1c, *PSA* prostate specific antigen, *PSM* positive surgical margin.

## Discussion

In the present study, we observed that the preoperative HbA1c level was significantly associated with worse clinical characteristics and adverse pathological outcomes in patients who were treated with RP for PCa. Furthermore, a high HbA1c level was found to be a statistically significant predictor for shorter postoperative BCR-free survival, whereas a previous history of DM did not show any significant associations. In addition to the oncological outcomes, the preoperative HbA1c level was associated with functional outcomes after surgery, specifically in the recovery of urinary continence. The recovery of erectile function was also better in the low HbA1c group, but the results were not statistically significant in this study.

Previously, DM has been reported to be associated with an increased risk of various cancers, including colo-rectum, breast, pancreas, endometrial, liver, and bladder^12–17^. However, the relationship between DM and PCa has been regarded as an inverse relationship^18–19^. The unique inverse association between DM and PCa development has not been fully understood, but previous studies suggested some possible explanations, including the detection bias from regular checkups, hormonal differences, and metabolic alterations associated with DM. Moreover, some studies demonstrated that DM was related to worse survival outcomes in PCa despite the inverse relationship between DM and PCa development^20–22^. Lee et al. performed meta-analysis with the data of 274,677 subjects from 17 cohort studies and found that there was a 29% increase in cancer-specific death in patients with DM (RR 1.29, 9% CI 1.22–1.38, I2 = 66.68%) than in others without DM^21^. On the other hand, other studies demonstrated no significant associations between DM and survival of PCa^23–25^. In the present study, we observed that the simple previous history of DM was not statistically related to postoperative recurrence after a surgical treatment for localized PCa. However, the status of preoperative glycemic control, which was represented by the HbA1c level, showed significant associations with postoperative oncological outcomes.

Not many studies have reported on the impact of glycemic control on the outcome of PCa independently with a history of DM. Gapstur et al. previously reported that hyperglycemia was significantly associated with increased mortality for PCa after analyzing 20,433 subjects who underwent health screening examination^26^. In addition, another study by Ma et al. found that men with high C-peptide (an insulin surrogate) had a > 2-fold increased risk of PCa-specific mortality than those with low C-peptide^27^. More recently, Farnoosh et al. analyzed 1,502 subjects who had DM history with HbA1c measurements before RP after analyzing the Shared Equal Access Regional Cancer Hospital database^28^. They found that high HbA1c was significantly associated with metastasis (HR 1.21, 95% CI 1.02. 1.44, *p* = 0.031) and progression to castration-resistant PCa (HR 1.27, 95% CI 1.03–1.56, *p* = 0.023). However, these studies investigated the relationship between HbA1c level and PCa outcomes only in patients with a history of DM but not in those without DM history. In the present study, we measured HbA1c as a routine preoperative work-up regardless of a previous history of DM and tried to evaluate the clinical influence of glycemic status on the postoperative outcomes. We observed that preoperative glycemic control was associated with postoperative oncological outcomes, but not with prior history of DM diagnosis.

It should be noted that the preoperative HbA1c level was also related to better urinary function recovery after surgery, in our study. We are not the first to evaluate the influence of DM and/or glycemic status on functional recovery after RP. Teber et al. previously reported that the history of type 2 DM was a strong predictor of postoperative incontinence in their retrospective analyses of 2071 patients after laparoscopic RP^29^. However, their study was limited by the small number of subjects, as there were only 135 patients with type 2 DM in the study. Considering that the current DM prevalence is approximately 10.5% for the overall population and even higher in the elderly, their study might have been biased due to some selection bias and/or recall bias. Furthermore, they performed a logistic regression test without considering the time interval between surgery and recovery of incontinence. In the present study, we compared the impact of glycemic control on postoperative incontinence with consideration of time onset for exact comparison. High preoperative HbA1c was revealed to be an independent predictor for worse recovery of postoperative incontinence, both as categorical and continuous variables. On the other hand, the recovery for erectile dysfunction was also superior in the low HbA1c group than in the high HbA1c group in our study, but the result was not statistically significant (*p* = 0.080). We believe that the influence of HbA1c on erectile dysfunction should be reevaluated in future studies because our study could not analyze a sufficient number of subjects who had normal erectile function before surgery.

We acknowledge that there may be limitations to our study, including the retrospective study design. Moreover, the main limitation of this study is that we could only analyze the level of HbA1c and not of the other hormones related to glycemic control, such as insulin or glucagon. Another limitation is that the glycemic control was only estimated by a single preoperative measurement, not by several postoperative follow-ups. As the glycemic status can vary according to the different time points and patients’ postoperative clinical status, the single measurement cannot be the exact representative for patients’ glycemic status. And more importantly, there is a possibility for selection bias in the present study as we only included the patients who were treated by radical prostatectomy. Therefore, patients who cannot tolerate the surgery for poor general condition or patients with locally-advanced disease were not included our study. Another limitation for our study is that we could not analyze the influence of salvage treatment after BCR as the salvage treatment can also affect the postoperative functional outcomes. Finally, we could not analyze the influence of usage for phosphodiesterase type 5 inhibitor and other general conditions which can affect the level of HbA1c. Therefore, our findings should be re-tested in future studies with prospective design and longer follow-up.

## Conclusions

A worse preoperative glycemic status, represented by the HbA1c level, was clinically associated with inferior oncological outcomes after surgical treatment of localized PCa. The recovery of urinary incontinence was also significantly inferior in the subgroup with worse preoperative glycemic status. On the other hand, the history of DM did not show any clinical association with oncological and functional outcomes after RP.

## Supplementary Information

Supplementary Table 1.
