# Supplementary material for: Impact of poor glycemic control upon clinical outcomes after radical prostatectomy in localized prostate cancer
Source: Sci Rep. 2021 Jun 7;11:12002. doi: 10.1038/s41598-021-91310-3 (PMC8184888; doi:10.1038/s41598-021-91310-3)
Supplement: Supplementary file 1 — Supplementary Table 1. [file 41598_2021_91310_MOESM1_ESM.docx]

| Supplementary Table 1. Univariate analyses using Cox proportional hazard model on biochemical recurrence | | | | |
| --- | --- | --- | --- | --- |
|  | When adjusted by history of DM | | | |
|  | HR | 95% CI | p value | |
| Age | 1.007 | 0.993 – 1.020 | 0.337 | |
| BMI | 1.003 | 0.982 – 1.024 | 0.765 | |
| History of DM | 1.164 | 0.942 – 1.439 | 0.159 | |
| PSA | 1.008 | 1.007 – 1.009 | < 0.001 | |
| HbA1c | 1.127 | 1.114 – 1.329 | < 0.001 | |
| Prostate volume | 0.999 | 0.993 – 1.006 | 0.867 | |
| Pathologic grade group |  |  |  | |
| Group 1 | Reference | | | |
| Group 2 | 13.070 | 3.239 – 52.731 | | < 0.001 |
| Group 3 | 42.530 | 10.292 – 175.746 | | < 0.001 |
| Group 4 - 5 | 127.465 | 31.365 – 518.016 | | < 0.001 |
| Pathologic stage (≥pT3) | 2.934 | 26.74 – 3.219 | | < 0.001 |
| PSM | 4.931 | 4.087 – 5.950 | | < 0.001 |
| Lymph node invasion | 9.135 | 6.888 – 12.117 | | < 0.001 |
| BMI, Body mass index; DM, diabetes mellitus; HbA1c, hemoglobin A1c; PSA, prostate specific antigen; PSM, positive surgical margin | | | | |
